# Smart Dressings Based on Nanostructured Fibers Containing Natural Origin Antimicrobial, Anti-Inflammatory, and Regenerative Compounds

**DOI:** 10.3390/ma8085154

**Published:** 2015-08-11

**Authors:** Vanesa Andreu, Gracia Mendoza, Manuel Arruebo, Silvia Irusta

**Affiliations:** 1 Department of Chemical Engineering, Aragon Institute of Nanoscience (INA), University of Zaragoza, Campus Río Ebro-Edificio I+D, C/ Mariano Esquillor S/N, 50018 Zaragoza, Spain; E-Mails: vandreu@unizar.es (V.A.); graciamendoza@gmail.com (G.M.); 2 Networking Research Center on Bioengineering, Biomaterials and Nanomedicine, CIBER-BBN, Madrid 28029, Spain

**Keywords:** dressings, chronic wounds, nanostructured materials, electrospinning, honey, essential oils, tissue engineering, infection, inflammation, regeneration

## Abstract

A fast and effective wound healing process would substantially decrease medical costs, wound care supplies, and hospitalization significantly improving the patients’ quality of life. The search for effective therapeutic approaches seems to be imperative in order to avoid the aggravation of chronic wounds. In spite of all the efforts that have been made during the recent years towards the development of artificial wound dressings, none of the currently available options combine all the requirements necessary for quick and optimal cutaneous regeneration. Therefore, technological advances in the area of temporary and permanent smart dressings for wound care are required. The development of nanoscience and nanotechnology can improve the materials and designs used in topical wound care in order to efficiently release antimicrobial, anti-inflammatory and regenerative compounds speeding up the endogenous healing process. Nanostructured dressings can overcome the limitations of the current coverings and, separately, natural origin components can also overcome the drawbacks of current antibiotics and antiseptics (mainly cytotoxicity, antibiotic resistance, and allergies). The combination of natural origin components with demonstrated antibiotic, regenerative, or anti-inflammatory properties together with nanostructured materials is a promising approach to fulfil all the requirements needed for the next generation of bioactive wound dressings. Microbially compromised wounds have been treated with different essential oils, honey, cationic peptides, aloe vera, plant extracts, and other natural origin occurring antimicrobial, anti-inflammatory, and regenerative components but the available evidence is limited and insufficient to be able to draw reliable conclusions and to extrapolate those findings to the clinical practice. The evidence and some promising preliminary results indicate that future comparative studies are justified but instead of talking about the beneficial or inert effects of those natural origin occurring materials, the scientific community leads towards the identification of the main active components involved and their mechanism of action during the corresponding healing, antimicrobial, or regenerative processes and in carrying out systematic and comparative controlled tests. Once those natural origin components have been identified and their efficacy validated through solid clinical trials, their combination within nanostructured dressings can open up new avenues in the fabrication of bioactive dressings with outstanding characteristics for wound care. The motivation of this work is to analyze the state of the art in the use of different essential oils, honey, cationic peptides, aloe vera, plant extracts, and other natural origin occurring materials as antimicrobial, anti-inflammatory and regenerative components with the aim of clarifying their potential clinical use in bioactive dressings. We conclude that, for those natural occurring materials, more clinical trials are needed to reach a sufficient level of evidence as therapeutic agents for wound healing management.

## 1. Introduction

**Figure 1:**
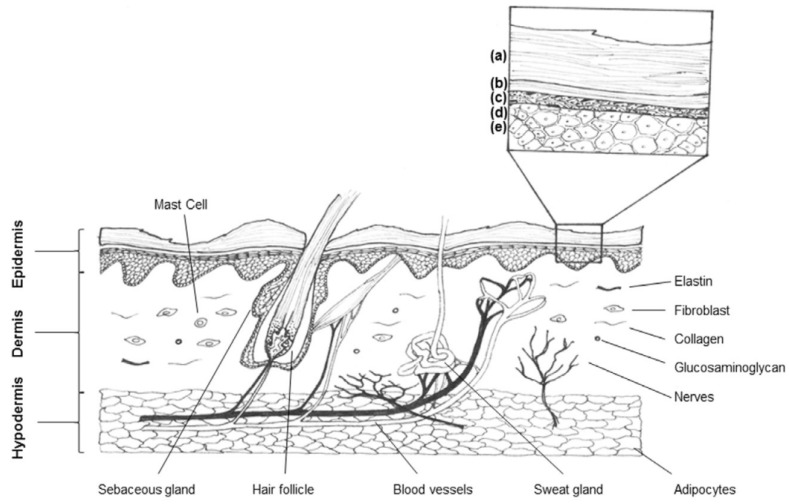
Schematic representation of human adult skin. Five different layers form the stratified epidermis: stratum corneum (**a**), stratum lucidum (**b**), stratum granulosum (**c**), stratum spinosum (**d**) to the stratum basal (**e**) [1]. (Copyright Karger Publishers 2015).

Human skin is the largest organ in our body. Adult skin comprises three different layers: the keratinized stratified epidermis, formed by the stratum corneum and by the underlying nucleated epidermis composed of four differentiated layers showing complex cell-cell interactions; the dermis which is a thick collagen-rich connective tissue where follicles, glands, nerves and capillary vessels are embedded; and the hypodermis which mainly consists of blood vessels and adipose tissue (Figure 1) [1,2,3]. It forms an effective barrier between the external environment and our organism protecting us from water loss and against pathogens and harmful assaults [4]. Its complexity comprises a wide number of interconnected constituents providing not only physical protection but also biochemical and adaptive immunity. Any change in these constituents results in the alteration of the barrier function, which can culminate in a potential damage [3,5].

This alteration or damage in the skin is of great importance as skin is our first protective barrier, so, a fast and efficient repair is imperative to avoid bacterial colonization, chronification, or even more severe injuries. When the wound healing machinery does not work properly, it is necessary to use a therapeutic treatment to speed up the natural process in order to avoid more serious pathologies and to improve the patients’ quality of life. These treatments can be as varied as the type of wound and as the part of the body to be healed, and constitute an enormous burden for any health care system. The search of cost-effective therapies for wound healing is becoming an essential topic of ongoing research and discussion worldwide.

### 1.1. Wounds

Skin wounds are described as disruptions in the normal anatomical structure of the tissue which lead to an abnormal behavior. One of the essential issues to study, diagnose, and treat a wound is its assessment. Wound assessment has different aspects but the most important is wound extent considering the extension of tissue damage and its severity. Skin wounds normally affect just the epidermis and dermis and are produced by internal or external forces. However, some wounds are or become deeper producing more severe outcomes including the hypodermis, fascia, muscle, tendon, bone and viscera, which are normally defined as ulcers [6,7].

In general terms, wounds may also be classified as acute and chronic wounds. Acute wounds are often produced by trauma (e.g., burns, lacerations, abrasions) and repair themselves in an organized and well timed process which results in the anatomic and physiologic re-establishment of the damaged skin [8]. On the contrary, chronic wounds fail time-wise, so a complete restoration of the skin structure and function is not successful in those cases [7,9]. Chronic wounds such as pressure ulcers are relatively common in long-term patients with reduced mobility, which may lead not only to skin damage but also to progressive injury into the muscle, tendon and bone, as well as to infection, septicaemia, osteomyelitis and, even death. Other ulcers, such as leg or venous ulcers, are also chronic wounds produced by venous hypertension or by failure in venous valves [9,10]. Foot ulcers and associated wounds are one of the most devastating complications of diabetes, often associated to injury and infections which may also lead to serious complications or even amputation due to the lack of sensitivity to injury, blister formation or cut [9]. Early detection is undoubtedly a key factor to avoid these complications together with an effective wound care.

### 1.2. The Clinical Burden of Wounds in the Health Care System

In the last decades, the increase in longevity, mainly in developed countries, due to medical breakthroughs and improved life quality, demonstrates a sign of progress, but also brings an increase in chronic health problems which, in several cases, implies the presence of non-healing wounds [11]. The chronicity in wounds has become a serious problem not only due to the economic burden of their clinical care but also to the emotional costs for patients and their families [7].

Concerning the financial burden for health care systems, wound complications are linked to bed occupation in hospitals, nurse time spent in wound care, longer and more intensive treatments, and medical and surgical interventions [10]. In this regard, the Eucomed Advanced Wound Care Sector Group recommended in 2008 to the European Commission the support and promotion of research in wound treatment and prevention in the light of the four million annual wound incidences in Europe [12]. The Canadian Association of Wound Care has recently published very interesting data [13] pointing out to an average cost of C$ 10,376 for treating a chronic wound and C$ 11,840 for treating an acute wound without complications, 165 days being the average time to closure for the later.

Pressure wounds have shown high prevalence in long-term patients in Europe (~20%) with the subsequent costs in pressure-redistributing equipment and additional nursing time. For example, it was estimated an annual burden of € 461 million in Spain in 2006 associated with those wounds, which represents the 5% of the total health-care spending, and, in 2009, it was estimated that the US spent US$ 11 billion in the management of these types of wounds [14]. In addition, in 2008, leg and foot ulcers showed an estimated annual burden of € 10–12 billion to the European Union health care systems. Furthermore, it has been calculated that in Europe, in a hospital performing 10,000 operations annually, 3%–4% of the surgeries lead to an infected wound with an annual cost of about € 2 million [10].

Even though the true impact of wound care can only be estimated, the data summarized above clearly show the enormous economic burden of wound care. A more rapid and effective wound healing process would substantially decrease medical costs, wound care supplies and hospitalization, and would significantly improve the patients’ quality of life.

### 1.3. Wound Healing Process: Stages of Wound Healing

**Figure 2:**
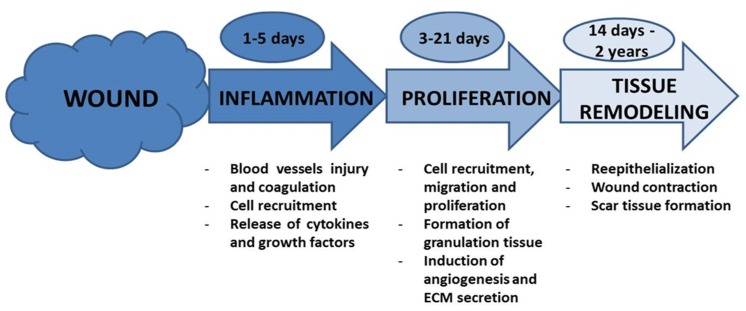
Normal wound healing stages with the main events involved.

**Figure 3:**
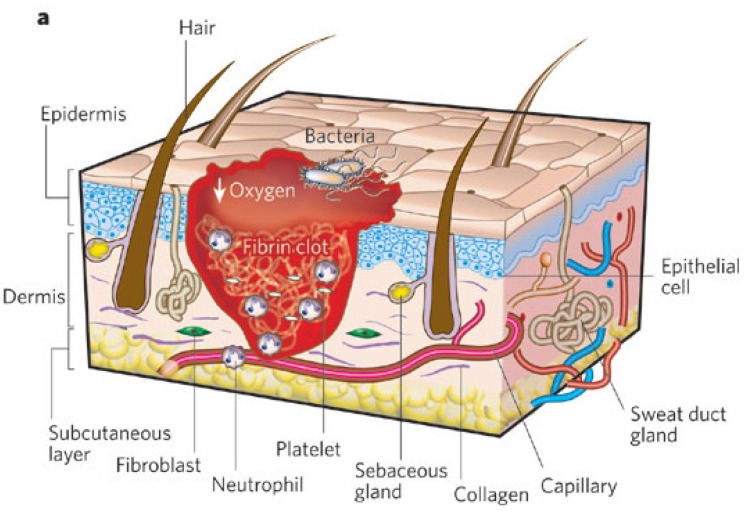

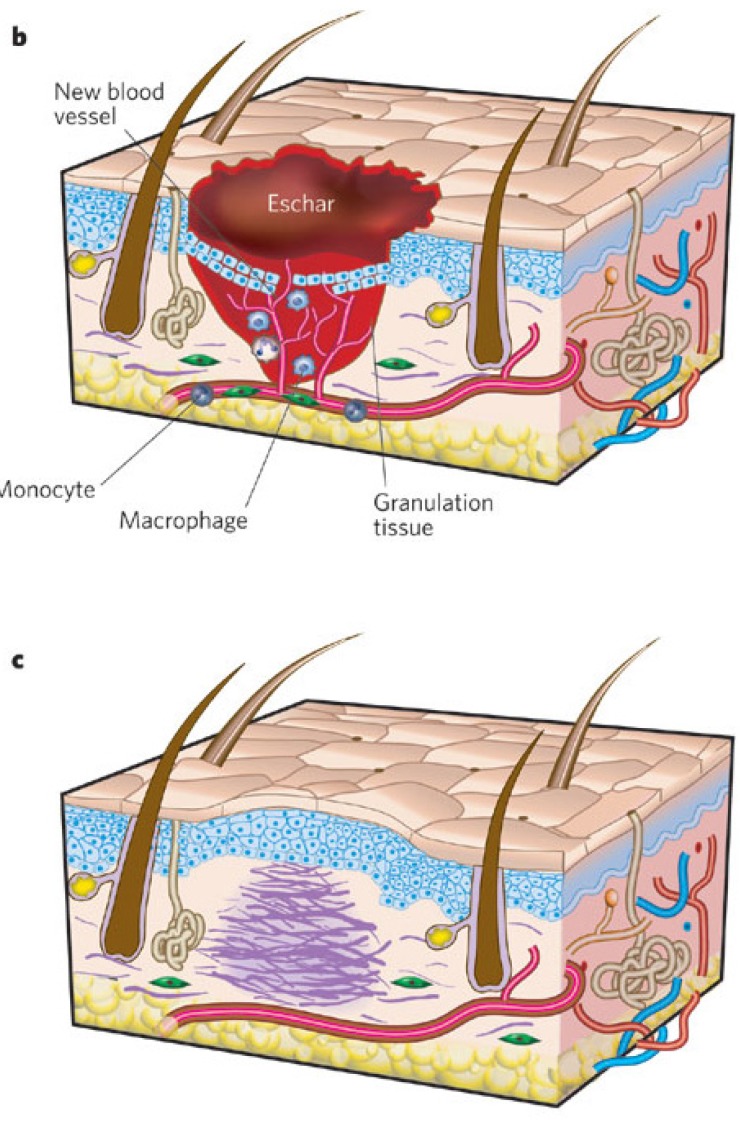
Wound healing stages: inflammation (**a**), proliferation (**b**) and tissue remodeling (**c**). (**a**) Inflammation is characterized by blood vessel injury, coagulation, and acute local inflammatory response with the formation of the protective clot against microbial infections; (**b**) The proliferation phase involves cell migration, granulation tissue formation, and angiogenesis; (**c**) Tissue remodeling implies the formation of a disorganized ECM as is depicted in the picture as well as a slightly elevated area together with the lack of normal skin appendages [22]. (Copyright Nature Publishing Group 2008).

Healthy epithelium maintains its homeostasis due to a complicated balance between tissue injury and regeneration which is highly regulated by epithelial stem cells [15]. Wound healing is a highly complex dynamic process which goal is the total restoration of the skin structure and function [1,16], and consists of three differentiated though overlapping phases: inflammation, proliferation, and tissue remodeling (Figure 2 and Figure 3) [2,17,18]. 

The inflammatory stage involves blood vessel injury, coagulation, and an acute local inflammatory response [6], with the recruitment of neutrophils, monocytes, macrophages, and lymphocytes as well as infiltration of leukocytes and subsequent secretion of inflammatory cytokines and growth factors [19].

In the proliferation phase, the cytokines and factors released in the previous stage stimulate the proliferation of progenitor cells, the recruitment of fibroblasts, keratinocytes, and endothelial cells, and finally cell migration and proliferation. As a consequence of these events, at this proliferative stage, granulation tissue is formed, angiogenesis induced and extracellular matrix (ECM) secreted [19,20,21].

The remodeling phase is characterized by the epithelial-mesenchymal transition (EMT) where cells migrate to re-epithelialize the damaged tissue in the edges of the wound. Then, wound contraction occurs and fibroblasts differentiate to myofibroblasts which results in scar-tissue formation [15]. As a consequence of the angiogenesis initiated in the previous stage, new blood vessels are generated followed by nerve sprouting [21].

Different factors may hinder those events including age, obesity, social causes, drugs or previous diseases [7,9,11,16,17,23]. Hypertrophic scars, which are the result of an excessive deposition of collagen, and non-healing (chronic) wounds are commonly present in the clinic [19]. Chronic wounds are frequently associated with a reduced number and well-defined pathologies, such as venous insufficiency, ischemia, diabetes mellitus, pressure necrosis, or vasculitis, which can affect healing at different stages. Several experimental clinical studies have clearly shown failure in the availability of growth factors to aid in the healing cascade because of insufficient production, defective release, trapping and/or increased degradation in those chronic cases [6,24]. On the other hand, angiogenesis may be insufficient due to previous pathologies [25,26,27,28].

However, not only previous diseases can hinder healing, the microenvironment of the wound may also play an important role. [16,18,19]. Bacterial components have been highlighted as harmful factors during wound healing due to their interference with cell-matrix interactions and due a reduced inflammatory response they produce [16,23,29,30]. As in other infective processes, bacteria can colonize wounds as a biofilm which is a complex aggregate of bacteria embedded in an ECM with the ability to form a highly resistant impervious microenvironment against antibiotics while maintaining the inflammatory stage [31,32].

## 2. Solutions and Standard Care Guidelines

### 2.1. Current Therapeutic Approaches/Options for Chronic Wound Treatment

The effective treatment of chronic wounds should be aimed at identifying and resolving the potential etiologic factors responsible for the non-healing wound state, leading to an important impact on the final clinical outcome. Several clinical practice guidelines and established medical protocols used during wound management have been extensively described to promote greater uniformity of care and to improve treatment outcomes. In that sense, the Wound Bed Preparation (WBP) emerged in 2000 to systematize the treatment of chronic wounds. It was defined as the global wound management in order to accelerate endogenous healing or to facilitate the effectiveness of other therapeutic approaches [33]. In that way, an initial assessment of the wound together with the overall health status of the patient, to consider existing pathologies which might hamper the endogenous healing, are indispensable for implementing an adequate treatment programme [34]. In addition, an evaluation of the wound state and response to the treatment should be regularly done.

After thorough evaluation of the patient and their wound, a local wound-care plan must be initiated. Consequently, the removal of devitalised tissue, a minimal amount of necrotic tissue, the maintenance of normal endogenous antimicrobial and proteolytic activity and an adequate control of inflammation and exudate are indispensable requirements for the progression of wound healing, and, therefore, the successful treatment of a complex wound.

Numerous techniques are employed nowadays for wound debridement. The choice of this debridement method do not depend on the diagnosis of the wound but on the presence of certain tissues covering the wound as well as on particular factors related to the patient and wound situation [35].

Inflammation is a physiological response to wounding and is required for wound healing to progress. However, excessive or inappropriate inflammation provides an ideal environment for bacterial infiltration and proliferation and may cause serious health problems. So, the prolonged inflammation characterizing the chronic wounds is a promising target for therapeutic interventions [36,37]. The recommended treatment for managing infection is a combined strategy to reduce the bacterial burden and to optimize the host response. In last years, there has been a renewed interest in the use of the antiseptics for the management of infected wounds due to the ongoing risk of allergies and resistance to topical and systemic antibiotics. The range of topical antiseptics agents currently used in the management of wound infection includes alcohols, acetic acid, chlorhexidine, honey, hydrogen peroxide, hypochlorous acid, potassium permanganate, polyhexamethyl biguanide (PHMB), products containing iodine (cadexomer iodine and povidone iodine) and products containing silver (silver sulfadiazine and silver-impregnated dressings). Dressings incorporating these antiseptics can successfully be used in topical management to reduce the load of a wide variety of pathogens [36,37,38].

Exudate production is a normal feature of healing wounds. However, when wounds produce insufficient or too much exudate, and/or the composition of the exudate is harmful, a wide range of problems can occur that ultimately delay healing, distress patients, and consume considerable healthcare resources [39]. In local wound management, dressings are the main option for managing exudate, being employed to increase, maintain, or reduce wound moisture. The final phase of wound healing is the epithelialisation. Debridement, control of inflammation, and moisture are essential factors of wound bed preparation that may stimulate the edge of the wound to migrate, but if this systematic approach produces a well-vascularized healthy wound bed which still fails to heal, additional therapies may be required. [40,41].

Alternatively, in patients whose ability to heal is compromised and in situations where skin coverage is inadequate, advanced therapies like bioengineered skin substitutes can be used to replace either temporarily or permanently the form and function of the skin, promoting the healing of the wound [41,42,43]. Although, these products have some advantages such as their availability in large quantities and negligible risks of immunologic issues, the main limitation is their expense. Besides, they still rely heavily on optimal wound bed preparation.

### 2.2. Wound Dressings

#### 2.2.1. Classification of Dressings: Passive, Interactive, and Bioactive Dressings

As aforementioned, one of the main medical options for the treatment of chronic wounds is the use of appropriate topical dressings which play an important role for correcting the underlying causes of non-healing wounds and, therefore, to aid during the healing process. In some situations, dressings are used as the definitive treatment, whereas in others they perform an important adjunctive role, being an intermediate step for other treatments.

Since ancient times, wound dressings have evolved from natural origin materials that simply covered and concealed the wound to modern materials specially designed to provide particular benefits.

Numerous categories and classifications of wound dressings have been published. Wound dressings are generally classified into three broad groups based on their nature of action: inert/passive, interactive, and bioactive. For many years, the mainstay of wound management was simply to protect the wound and all therapeutic efforts focused on drying the wound site. For that, passive dressings which are ordinary dressings, such as gauze and tulle, merely were used to cover and conceal the wound while repairing underneath. In addition, these passive products, which have a minimal role in the healing process, prevented infection by forming a barrier against bacterial colonization. However, these dressings produce a waterproof cover over the wound and their use can lead to skin maceration around the wound since water vapour and exudation may not pass through becoming trapped within the wound [44]. This lack of vapour permeation results in dressing leakage and the need for frequent replacements. The pioneering idea to change the nature of wound dressing materials was the discovery of the critical role of moisture in wound healing proved by Winter in 1962 [45]. Moist wound healing refers to the provision and maintenance of optimal hydration of the exposed tissues in the wound, establishing and maintaining an optimal environment for wound repair. Thus, the newer dressings have been designed to establish and maintain an optimal environment for wound repair. Interactive dressings are capable of modifying the physiology of the wound environment and interact with the wound surface to optimize healing by promoting debridement, enhancing granulation and re-epithelialization, and reducing the exudate levels and bacterial colonization counts. Interactive products are mostly transparent, permeable to water vapor and oxygen and impermeable to bacteria [46,47,48]. Some of the products including in the category of interactive dressings are hydrocolloids, alginates, collagen, hyaluronic acid (HA) products, foams, hydrogels, and semipermeable films.

In spite of these dressings, many wounds still persist. In order to promote the healing in these complex wounds, numerous bioactive dressings have been developed. These products deliver active substances, such as antimicrobials and antibiotics, which have a direct role in changing the chemical and cellular environment of the local wound, stimulating the healing cascade [48,49].

#### 2.2.2. Desirable Characteristics of Wound Dressings

Nowadays, an ideal wound dressing material should have extraordinary properties which would enhance/improve the wound healing process including its ability for maintaining a moist wound environment, debridement of wound site, absorption of excess exudate and blood at wound site, free of particles and toxic wound contaminants, non-toxic and non-allergenic, allow an adequate gaseous exchange, capable of protecting the wound from further trauma, low adherence and ease of removal, infection prevention and bacterial invasion protection, thermal insulation, minimal frequency of dressing change, easy of application, long shelf-life, comfortable and conformable, and cost effectiveness [47,50,51].

However, in spite of all the efforts that have been made during the last years towards the development of artificial wound coverings, no currently available dressing combines all those previous requirements necessary for a quick and optimal cutaneous regeneration. Therefore, more technological advances in the area of temporary and permanent smart dressings for wound care are required.

## 3. Materials

### 3.1. Nanostructured Dressings

Current efforts in tissue engineering are focused on the creation of three-dimensional scaffolds mimicking the ECM. However, when the tissue does not have its inherent potential to regenerate, the supply of only a scaffold to the defective site does not assure tissue repair. In that way, as an additional method of tuning the biomimetic artificial matrix, different biological molecules such as growth factors, angiogenic factors, cell surface receptors and antimicrobial, antibacterial, and anti-inflammatory agents can be easily incorporated into those scaffolds to improve cell behaviour as well as tissue regeneration.

**Figure 4:**
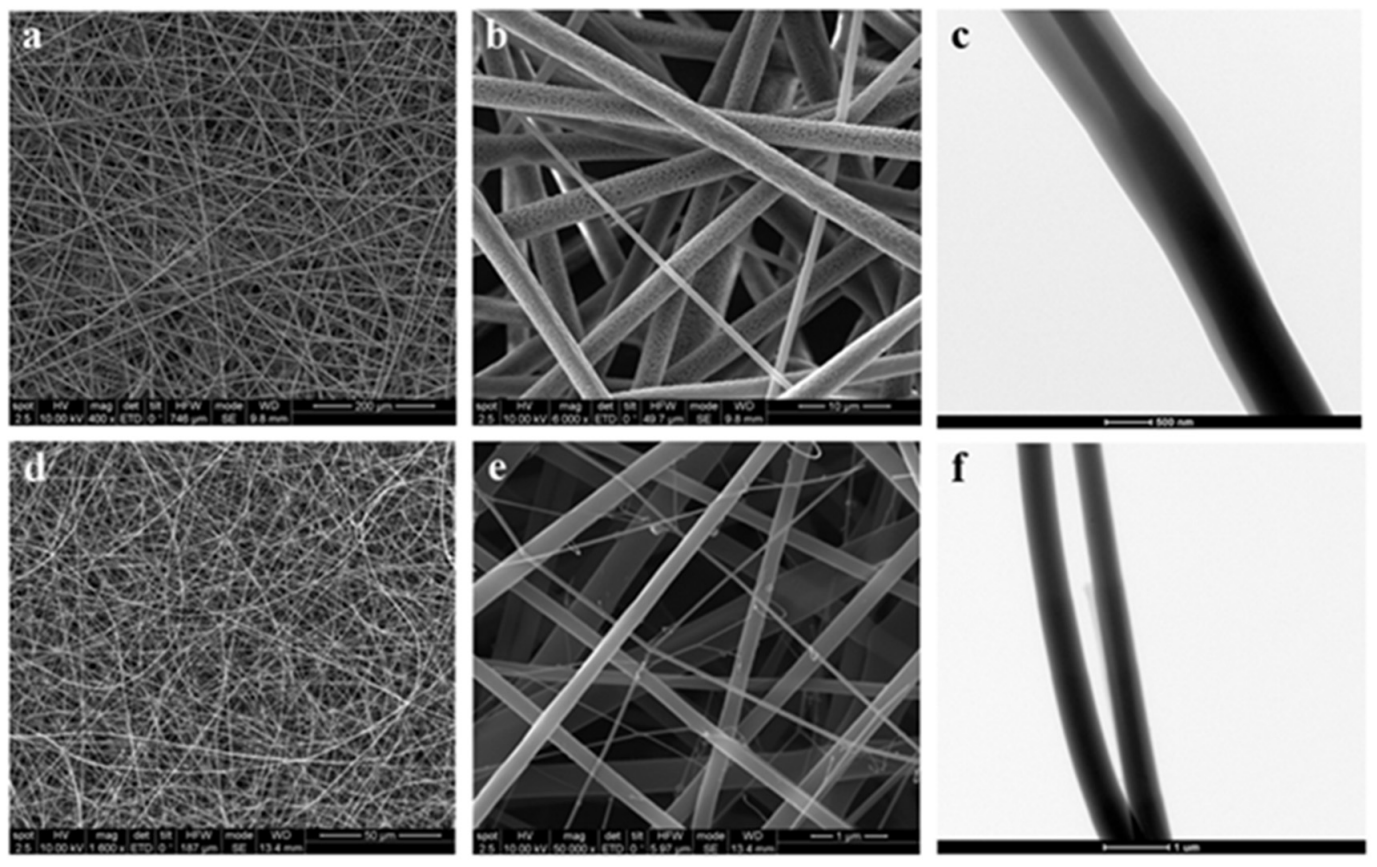
Scanning Electron Microscopy (SEM; **a**,**b**,**d**,**e**) and Transmission Electron Microscopy (TEM; **c**,**f**) micrographs of nanofibrous scaffolds, consisting of polylactic acid (PLLA)/polycaprolactone (PCL) (**a**,**b**) and chitosan (**d**,**e**) polymers, synthetized by electrospinning technique (unpublished results).

A wide range of nanoscale materials have been reported in the literature showing their successful application in tissue repair and wound management. Among all the nanoscale structures used to generate artificial ECM, nanofibrous scaffolds have been proposed as the most promising candidates (Figure 4) [52]. Several intrinsic properties of nanofibrous scaffolds, such as high surface area to volume ratio, sufficient mechanical stability and adequate pore size of the resulting nanofibrous matrix, make these materials particularly interesting for wound healing and drug-delivery applications. So, the high porosity of the nanofiber matrix allows oxygen and water permeability and nutrients exchange and also the removal of metabolic waste preventing fluid accumulation at the wound site. Furthermore, the high surface area favours cell attachment and subsequent proliferation and differentiation during tissue regeneration.

### 3.2. Scaffolds Based on Natural Origin and Synthetic Polymers

The efficacy of these replacements is widely dependent on the choice of their construction materials, structures and physicochemical properties. Polymers have been widely used as biomaterials for the fabrication of tissue-engineering scaffolds. Thus, different forms of scaffolds such as films, gels, sponges, membranes, micro and nanofibers made of natural origin and synthetic polymers have been developed and evaluated as dermal substitutes to date.

#### 3.2.1. Natural Origin Polymers

Natural origin polymers have been widely used in regenerative medicine because of their biocompatibility, biodegradability, biological characteristics and structural similarities with human tissues. Naturally derived materials from animals or plants, which are usually constituted by proteins or polysaccharides, mimic the fibrillary structure of native ECM and have similar architectural resemblances [53,54].

Collagen is the major protein component of the ECM, and multiple scaffolds based on this material have been described as building material in many biomedical applications including wound dressings. For example, collagen-minocycline based hydrogels are potentially applicable for the treatment of cutaneous wound infections [55], denatured collagen microfiber scaffolds seeded with human fibroblasts and keratinocytes can be used as skin grafts [56], collagen-alginic acid can also be used as wound dressing materials [57], electrospun collagen nanofibrous scaffolds have been also applied in wound repair [58]. In that regard, Powell et al. [59] compared collagen nanofibrous scaffolds produced by freeze drying and electrospinning methods as skin substitutes demonstrating that although both scaffolds can be used to fabricate skin substitutes with optimal cellular organization, proliferation and maturation, electrospun scaffolds can potentially reduce wound contraction much faster compared to the freeze-dried ones. Currently, Biobrane^®^, Integra^®^, Apligraf^®^, and Transcyte^®^ are available as commercial biological skin substitutes based on collagen and are widely used in wound healing [60,61].

Gelatin, a natural origin polymer derived from collagen, has also been used in medical applications, especially in the production of biocompatible and biodegradable wound dressings. The optimal morphology of an electrospun gelatin scaffold for skin repair was studied by Powel and Boyce [62] demonstrating that, principally, porosity and interfiber distance play a significant factor in tissue morphogenesis. More recently, Bilgic *et al.* [63] demonstrated that gelatin-based biodegradable scaffolds have an enormous potential to enhance wound healing.

Fibrin, a complex network naturally formed by fibrinogen polymerization in the presence of the enzyme thrombin, has been used widely as a natural origin scaffold for wound healing and tissue engineering applications owing to its characteristic advantages including reduced inflammation, immune response, toxicity, and enhanced cell adhesion [64].

The major drawbacks associated with the use of protein based-polymers are their low mechanical stiffness and rapid degradation rate *in vivo*. In order to overcome these disadvantages, crosslinking techniques, including chemical and physical methods, have played a pivotal role for producing scaffolds with enhanced mechanical properties as well as means of making water insoluble scaffolds [53,54,65]. For example, Ulubayram *et al.* [66] optimized the effect of the type and the amount of cross-linker used on the thermal and mechanical properties, stability, and cytotoxicity of gelatin sponges. More recently, genipin and glyceraldehyde have been used as crosslinkers due to their low toxicity and reproducibility in the creation of gelatin-based scaffolds [67,68]. Furthermore, a range of various cross-linkers (transglutaminase, genipin, EDC/NHS, and UV light exposure) were tested in electrospun collagen nanofibers to avoid the poor water resistance of collagen [69]. Additionally, the combination with other artificial scaffolding materials could resolve this handicap as well. The addition of inorganic fillers in the polymeric structure is also another alternative to increase the required mechanical stability under operation rendering nanocomposites with superior properties.

In regard to polysaccharide-based polymers, alkaline (chitin, chitosan), neutral (glucans, dextrans, cellulose), acidic (alginic acid, HA) or sulfonated polysaccharides (heparin, chondroitin, dermatan sulfate, keratan sulfate) are broadly used for the management of wounds. In that sense, films, gels, or sponges of chitosan have recently been investigated for their use in wound care, demonstrating antimicrobial and wound-healing effects [70]. In a parallel study, Tchemtchoua *et al.* [71] showed that the adhesion, growth, and differentiation of the three main skin cell types (keratinocytes, fibroblasts and endothelial cells) were enhanced when using chitosan nanofibrous scaffolds in comparison to other types of chitosan-based structures (films, sponges or gels). The natural polysaccharide alginate has also been proposed as suitable building materials in wound dressings. There are numerous studies concerning the application of different alginate-based formulations: chitosan-fibrin-sodium alginate composite for wound dressings [72], alginate nanofiber-based wound dressings [73], sodium alginate/poly(ethylene oxide) blend nanofibers [74], composite alginate, and gelatin-based bio-polymeric wafers for wound healing [75] and so on.

An important natural origin biopolymer of increasing interest is HA which assists in providing wound healing. Researchers have developed scaffolds based on HA for difficult-to-heal wounds, post-traumatic, and complicated surgical wounds [76,77,78]. Recently, gelatin-chondroitin-6-sulfate-HA [79], HA/Agarose [80] and photocrosslinked HA/fibronectin [81] based scaffolds have shown a great potential in wound healing scenarios.

#### 3.2.2. Synthetic Polymers

The functions and properties (e.g., porosity, degradation time, and mechanical characteristics) of synthetic polymers can be tailored by modifications in the synthesis processes according to specific requirements and applications in order to overcome many of the presented shortcomings of natural origin polymers. So, the variation of the different parameters such as chemical composition, crystallinity, molecular weight, and copolymerization allow production of scaffolds with predictable and reproducible mechanical and chemical properties in large quantities. In addition, they are often cheaper than biological scaffolds and have a longer shelf life.

FDA approved-aliphatic polyesters such as PLA, polyglycolic acid (PGA), PCL and their copolymers have being widely applied in skin tissue engineering and as wound dressings. In this regard, the development of several wound healing dressings such as curcumin-loaded PLA nanofibers [82], PGA/collagen composite nanofibrous scaffolds [83], nanofibrous scaffolds containing gum tragacanth/PCL [84] and poly(lactide-co-glycolic acid) (PLGA)/silk fibroin hybrid scaffolds [85] have demonstrated a great potential as wound healing materials. Sun *et al.* [86] showed that the use of ginsenoside Rg3-loaded electrospun PLGA fibrous membranes as wound covers induces healing and inhibits hypertrophic scar formation.

Other commonly synthetic polymers used as wound dressing include polyurethane (PU), polyvinyl alcohol (PVA), polyethyleneoxide (PEO) and polyethyleneglycol (PEG). For example, Yari *et al.* [87] analyzed the application of different cross-linked PU hydrogels with chemically anchored antibacterial groups as wound dressings. Besides, electrospun PU-dextran nanofiber mats containing ciprofloxacin HCl showed enhanced antibacterial activity [88]. Also, Dai *et al.* [89] demonstrated that electrospun emodin polyvinylpyrrolidone (PVP) blended nanofibrous membranes accelerated wound healing compared to standard treatments. An *in vivo* study carried out by Yun *et al.* [90] demonstrated that the use of a fibroblast-encapsulated PEG-b-poly(L-alanine) thermogel significantly improved the healing-process speed and also the dermal regeneration compared to control systems based on just cell-free PEG-b-poly(L-alanine) thermogel and PBS. In another study, it was demonstrated that PEG incorporation into ciprofloxacin hydrochloride loaded chitosan scaffolds was effective for quicker and regulated wound healing [91].

### 3.3. Fabrication (Electrospinning, Phase Separation, Auto-Assembly, etc.)

A wide variety of techniques have been used to fabricate polymers into different types of nanostructured dressings in order to improve the wound healing processes and to ensure a good efficient drug loading. The fabrication of these dressings should be strictly controlled to obtain the appropriate features to perform a successful and rapid healing. In fact, porosity is a key factor in the promotion of new tissue formation because cell migration, proliferation, and differentiation are all affected by geometrical constraints from the surrounding microenvironment, and it should be adapted for each application [92,93,94,95]. However, the required porosity depends on the proximity of capillary vessels to irrigate the wounded tissue. If the wound is close to a very well irrigated area the need of generating new angiogenesis, and the corresponding large pores, is reduced, but in less irrigated areas large pores are required in the scaffold to allow new capillary generation. Also the size of the remodeling zone is important to consider when the appropriated pore size scaffold is to be selected. Furthermore, parameters linked to porosity such as interconnectivity or changes mediated by degradation or remodeling should be carefully monitored [96,97].

**Figure 5:**
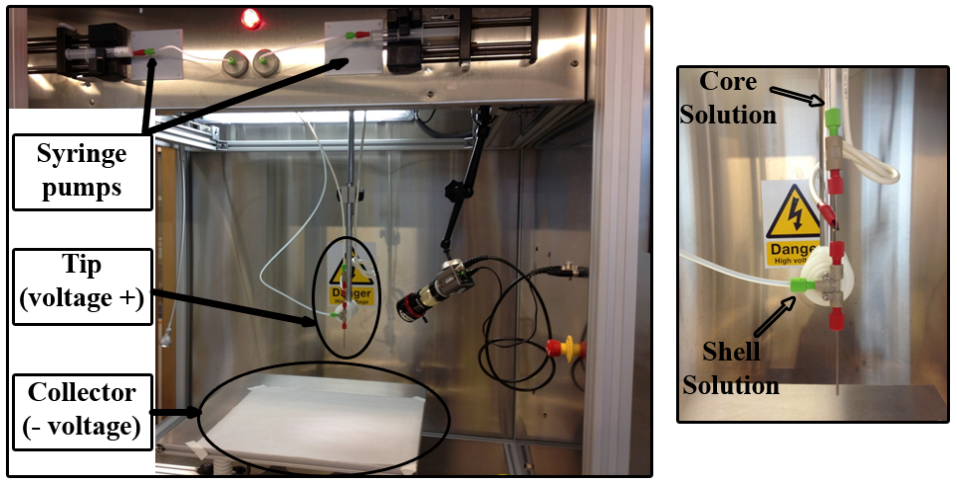
Electrospinning experimental set-up with its main components: syringe pumps, tip and collector. Coaxial injection capillaries can be used (image on the right) to fabricate nanofibrous core-shell structures.

In the last few years, one of the most extended methods for the fabrication of nanodressings is the electrohydrodynamic (EHD) technique or electrospinning. EHD techniques use electrostatic forces to produce from nano- to microstructures composed of continuous ultrafine fibers rendering multiple shapes due to the use of an electrically charged fluid stream which is collected in its corresponding counter-electrode the applied voltage being a crucial element in the formation of the fibers [98,99,100]. The features of the resulting structures regarding composition or morphology can be modified through the type of polymer or solvent used or even by modifying the injection speed, in order to fulfil the requirements for the envisaged applications which are diverse such as biomedical, pharmaceutical, or security and defense [98,101,102]. This simple technique has become very popular for the fabrication of these fibers because of its potential to easily scale up or the capability to spin very different types of polymers and also the simplicity and low-cost of the equipment required. The fiber is formed when after the application of a high electrical potential to a polymer droplet at the tip of an injection needle this is ejected when repulsion charges overpass surface tension. Finally, the solvent is evaporated in its way towards the grounded counter-electrode and fibers are collected (Figure 5) [102,103].

Electrospun fibers are widely used in dressings and in drug delivery applications due to their specific features such as high area to volume ratio, customized porosity, morphology, and functionalization which can provide them with hemostatic ability, protection against infections, adequate maintenance of moisture or even mimicking the ECM [98,101,104,105,106]. Very different polymers have been used to produce these fibers from natural origin polymers (e.g., chitosan, collagen, HA), to synthetic polymers (e.g., PLLA, PCL) and copolymers (e.g., PLGA, poly(l-lactide-co-caprolactone)) in order to produce scaffolds, membranes or implants for tissue engineering and/or with drug delivery ability [98,100,103,107,108,109,110,111,112,113]. Specifically, electrospun PLLA/PEG materials exerted *in vitro* bactericidal and hemostatic effects [114] while electrospun collagen dressings have shown better *in vivo* healing ability than classic wound care systems [115]. Moreover, silk fibroin/PLGA electrospun nano-dressings displayed improved wound closure compared to just PLGA dressings and to control samples in an *in vivo* wound model of diabetic rats due to a demonstrated improved re-epithelialization process [85].

In addition, other techniques have been developed for the fabrication of nanofibers with biomedical purposes and high drug loading capacity though less extended than electrospinning. The phase separation technique involves the polymer dissolution, gelation, and solvent extraction followed by freeze drying or vacuum. Under specific conditions, a homogeneous polymer can be separated into two phases due to thermodynamic instability or even by mechanical shearing or emulsification of two or more phases, to finally obtain a solid polymer-rich phase. The structural characteristics of the final product can be modified changing the thermodynamics and kinetics of the specific reaction. In addition, this technique allows the loading of sensitive drugs without loss in their bioactivity [92]. Moreover, self-assembly is another technique to produce nanofibers which results in the formation of a nanofibrous mesh through the organization of preexisting components [116]. Fiber mesh and fiber bonding are other methodologies that imply the deposition of a polymer over another polymer followed by an evaporation stage, and the joining of nanofibers at their cross-linking points in order to produce nanofibrous meshes [92,117]. 

In general, those techniques allow an easy, not expensive and vast production of nanofibers with high surface area, though the resulting structures, in general, do not show the required mechanical stability making control of the porosity difficult in the case of using phase separation, fiber mesh, and fiber bonding techniques; the resulting shapes are not as uniform as in self-assembly techniques, and the loading efficiency is low when using self-assembly or fiber mesh techniques [99,116,117].

### 3.4. Dressings for Controlled Drug Delivery to the Wound

Drug delivery systems are biocompatible particles, hydrogels, dressings, or three-dimensional (3D) devices that are able to provide sustained doses of a therapeutic moiety in our body accomplishing increased safety and efficiency due to the tight control of the site of release, dosage, and timing [118,119,120]. In the last years, a great number of nanostructured materials have been developed for drug delivery because of the increased drug-dissolution rate of these structures through the superior surface area per volume ratio [103].

Bioactive wound dressings in different forms (e.g., hydrogels, films, electrospun fibers) have been developed in recent years to improve the protection against bacterial infections [121]. These dressings should fulfill some requirements to develop their functions properly such as maintain moisture, favor hemostasis and cell proliferation, and allow functionalization and good flexibility [103]. As mentioned above, electrospun fibers are being widely used to fabricate nanostructured dressings with drug delivery ability. Furthermore, biomacromolecules as well as hydrophilic and hydrophobic drugs can be directly encapsulated into electrospun fibers making their use as drug release systems easy. These features together with their high surface area and 3D porous structure confer them with a high efficiency for drug delivery [101,102,122,123]. In addition, the local delivery provided by these types of fibers offer the possibility to reduce the minimum required drug dosage which implies less side effects [103].

The methodology to load a nanofiber with a specific drug should be highly specialized because drugs show different chemical characteristics and every disease needs the most suitable delivery system [103]. There are a wide number of techniques to achieve this goal by combining the most used. This methodology involves one-step electrospinning due to the dissolution or dispersion of the drug in an appropriated solution of the polymer [119,124]. Although it is a very simple technique, it is of great importance to know the drug distribution into the fibers and their possible interactions and drug kinetics to achieve the required release. Some researchers have improved this loading technique by adding hydrophilic polymers or amphiphilic copolymers or even by increasing the hydrophilicity of the fibers to obtain a higher loading efficiency and improved sustained release [121,125,126,127]. There are also other techniques used for drug loading in electrospun nanofibers in order to improve sustained delivery and maintain drug chemical properties, such as the modification of the fiber surface by bounding or conjugation of the drug to the surface of the nanofibers which is useful for achieving a slow release of therapeutic biomolecules (e.g., growth factors, genetic material) [102,103,128]. A modification of the electrospinning process called coaxial electrospinning in which the drug is placed in the inner jet and the polymer is in the outer tubing is also used to achieve a long term drug release [129]. The emulsion of the drug within the dissolved polymer or the sequential electrospinning to load more than one drug for combination therapies are also strategies widely used [101,103].

In this sense, several authors have shown very different studies in which electrospinning is the technique of choice to fabricate drug delivery nanodressings. A wide variety of materials have been used such as PLGA, poly(vinyl acetate), or cellulose, together with different drugs such as antibiotics, anti-inflammatory, antioxidants and/or natural origin compounds, demonstrating a higher efficiency than drug-loaded films [121,130,131,132]. For instance, Choi *et al.* [102] have shown in a mice diabetic ulcer model that the use of PCL or PCL-PEG nanodressings loaded with epidermal growth factors (EGF) clearly outperformed the healing of wounds compared to the nanodressings alone or with the non-treated wounds. In addition, these authors confirmed the beneficial effects of this type of dressings in keratinocyte expression in an *in vitro* model. Other researchers [133] have developed an *in vivo* wound healing model in diabetic rats highlighting the rapid and efficient restoration of the skin structure and function when wounds were treated with coaxially fabricated electrospun fibers loaded with basic fibroblast growth factor (bFGF). The gradual release of the growth factor resulted in higher capillarization, collagen deposition, and ECM remodeling in a similar fashion compared to normal skin. Furthermore, they also showed *in vitro* enhanced cell adhesion, proliferation and secretion of ECM in a mouse embryo fibroblast model.

Other researchers have focused their research on modifying textile dressings with magnetic nanostructures to prevent the evaporation of the natural origin compounds limonene and eugenol or other essential oils embedded in the dressings to achieve sustained bactericidal ability against different strains of bacteria [134,135].

There are also studies regarding the production of drug-eluting porous structures for wound dressing through the methodology of freeze drying of inverted emulsions [92,99]. In this regard, Elsner *et al.* [92] stated that drug loading can be performed with this technique at any stage of the fabrication without any drug loss or modification due to temperature or chemical interactions. In this case, drug release was modulated through the degree of porosity which in turn may be modified by controlling the organic:aqueous ratio or drug content. This study showed the high efficiency of these nanodressings loaded with the antibiotics gentamicin and ceftazidime against *Staphylococcus aureus*, *Staphylococcus albus* and *Pseudomonas aeruginosa*, both *in vitro* and *in vivo*, to improve wound healing processes.

## 4. Essential Oils, Honey, Aloe Vera, Cationic Peptides and other Natural Origin Antimicrobial, Anti-Inflammatory and Regenerative Compounds

This section encompasses the literature published in the last years describing the application of naturally occurring antimicrobials, anti-inflammatory and regenerative molecules in the acceleration of the wound-healing process. A wealth of *in vitro* and *in vivo* studies with animal models demonstrates the antimicrobial, anti-inflammatory, and regenerative properties of essential or edible oils, honey, aloe vera, plant extracts, cationic peptides, *etc.*; however in the sake of concision we shall only review those results which involve clinical trials. Results from enteral administration of these natural origin components have also been excluded in this review.

### 4.1. Antimicrobials

Everything has antimicrobial properties depending on its dose. “*The dose makes the poison*” is the basic principle of toxicology (credited to Paracelsus). The idea behind this section is to be thought-provoking, trying to compare the potential benefits from natural-origin materials topically applied against pathogenic microorganisms colonizing wounds compared to the conventional synthetic topical antiseptics (iodine, silver, chlorhexidine, polyhexamethylbiguanide, *etc.*) or with the systemic application of antibiotics and also always keeping in mind the required dose needed to reach the same antimicrobial level. It is important to mention that to prevent wound infection the use of topical antibiotics is not recommended due to the risk of sensitization and development of resistance. Natural origin materials have been used in wound care with positive results.

Natural origin materials can be antimicrobials or can boost the defenses of the infected host. As we mentioned before, when the antimicrobial is applied on the wound or on intact skin they are called antiseptics [136]. Obviously the material of choice to accelerate the physiological wound healing process must have antimicrobial action on bacteria, yeast, fungi, virus, and spores but should be non-cytotoxic on human cells without the development of any antimicrobial resistance.

Honey has been used as antimicrobial for centuries. The composition of the honey depends on the floral source that the honeybees use and also on the environmental conditions [137]. Its antimicrobial action is attributed to its acidic pH, high osmolarity, and to the presence of hydrogen peroxide (reactive oxygen species (ROS) generation), antioxidants, lysozyme, polyphenols, phenolic acids, flavonoids, methylglyoxal, and bee peptides [137]. A prospective, multicentre, open label randomized controlled trial with 808 patients with venous leg ulcers having ≥50% wound area covered in slough not taking antibiotics or immunosuppressant therapy were recruited to compare the efficacy of manuka honey versus current hydrogel therapy [138]. The results showed that patients treated with the manuka honey had increased incidence of healing, effective desloughing and a lower incidence of infection than the controls. However, Jull *et al.* [139] carried out a community-based open-label randomized trial on patients with venous ulcers using either calcium alginate dressings impregnated with manuka honey (187 patients) or usual care (181 patients) and showed that honey-impregnated dressings did not significantly improve venous ulcer healing at 12 weeks compared with the usual care. Another prospective open label multicenter study with 108 patients with sloughy venous leg ulcers treated with manuka honey or hydrogel showed a benefit of one versus the other depending on the bacteria present [140]. A randomized study with 69 patients using honey-coated bandages compared with silver-coated bandages on the treatment of malignant wound showed no differences between both groups [141]. In a prospective randomized study (45 subjects) to compare the effectiveness of honey dressings vs. povidone iodine dressing in chronic wound healing, honey dressings showed a significant decrease in the wound surface area, pain score and increase in comfort score compared to the iodine-based ones [142]. Shorter times of healing and a rapid disinfection of neuropathic diabetic foot ulcers were observed when using manuka honey-impregnated dressings compared to conventional dressings in a randomized clinical trial (63 patients) [143]. Literature reports varied results for honey depending on the infected wound [144] and also it has been demonstrated that honey may even have a detrimental effect on diabetic ulcers [145]. These contradictory results indicate that more information is needed and multi-center clinical trials and with a larger number of patients are required to show a clinical benefit for honey to reduce the incidence of wound infection [146].

Essential oils are composed of 20–80 constituents existing at significantly low concentrations in plants which chemical composition depends on climatic, seasonal, geographic conditions and distillation technique [147]. Their antimicrobial action is attributed to some of their varied components including terpenes, low molecular weight aliphatic hydrocarbons, acids, alcohols, aldehydes, acyclic esters, *etc.* Different edible oils extracted from fruits have antimicrobial action which is attributed to their acidic pH and to the presence of simple phenols and oxygenated compounds. The great advantage is that essential oils show little impact on the development of antimicrobial resistance and susceptibility compared to other biocidal components [148,149].

Darmstadt *et al.* [150] demonstrated that premature babies (159) who received a daily massage with sunflower-seed oil were 41% less likely to develop nosocomial infections than controls (without any treatment) and Aquaphor (petrolatum, mineral oil, mineral wax, lanolin alcohol; 157 babies) did not significantly reduce the risk of infection.

A randomized, controlled trial of tea tree topical preparations versus a standard topical regimen for the clearance of methicillin-resistant *S. aureus* (MRSA) colonization was carried out by Dryden *et al.* [151]. In this study 114 patients received standard treatment and a 49% of them were cleared of MRSA carriage. 110 patients received tea-tree oil regimen and only a 41% of them were cleared. The authors concluded that there was no significant difference between treatment regimens and, from the same study they also concluded that tea-tree treatment was more effective than chlorhexidine or silver sulfadiazine at clearing superficial skin sites and skin lesions. A recent prospective, open-label, randomized, controlled trial with 445 patients was carried out to determine whether the daily use of 5% tea-tree oil body wash compared with standard care had a lower incidence of MRSA colonization [152]. The results showed that a 10% of the patients developed new MRSA colonization and therefore, compared with standard care, the daily use of 5% tea-tree oil body wash cannot be recommended as an effective means of reducing MRSA colonization.

Essential coriander oil was very efficient as an antiseptic for the prevention and treatment of skin infections with Gram-positive bacteria (*Streptococcus pyogenes*, *S. aureus* and MRSA) [153]. In addition, no skin irritation could be observed by sensitive photometric assessment in any of the 40 volunteers using patches impregnated with this essential oil.

Lavender oil was used in the treatment of recurrent aphthous ulceration by means of randomized double-blind, placebo-controlled study performed in animal models and also in 115 subjects [154]. A significant ulcer-size reduction, increased rate of mucosal repair, and healing within three days of treatment were observed in the animals treated with lavender oil compared to baseline and placebo groups. Lavender oil showed a broad antibacterial activity against different tested strains and the patients treated with lavender oil showed a significant reduction in inflammation level, ulcer size, healing time, and pain relief. As in the case of honey-based products, the literature compiles contradictory or at least insufficient results to make a scientific conclusion about the demonstrated benefit of those essential oils.

Antimicrobial peptides are an evolutionarily conserved component of the innate immune response found among all classes of life ranging from prokaryotes to humans which show antimicrobial activities against Gram-positive and Gram-negative bacteria [155]. They act with multiple roles as mediators of inflammation with the effects on epithelial and inflammatory cells, influencing cell proliferation, wound healing, cytokine/chemokine production and chemotaxis [156]. They also do not seem to propagate the development of antibiotic-resistant micro-organisms [157]. Lipsky *et al.* [158] described the results of two consecutive, double-blind, randomized controlled trials on diabetic patients with a mildly infected diabetic foot ulcers to receive an active topical antimicrobial peptide, pexiganan acetate cream, or an active oral antibiotic (ofloxacin), plus a respective inactive placebo. The results with a total of 835 subjects showed equivalent results (within the 95% confidence interval) for topical pexiganan and oral ofloxacin in clinical improvement rates, overall microbiological eradication rates, and wound healing rates. A significant reduction of infectious complication after major liver surgery was also observed in a clinical trial with patients using bactericidal/permeability-increasing protein (rBPI (21)) [159].

Despite some of those good results, the main drawback is their high cost and also that it is difficult to maintain a constant optimal therapeutic level due to the short half-life of recombinant proteins *in vivo* [160].

Several pathogenic bacteria are developing antibiotic resistance and therefore, initially the study of the potential applicability of those natural occurring antimicrobial agents is justified [161,162]. However, as in the case of nanosilver, more studies are need to assess their potential role in antimicrobial resistance [163].

### 4.2. Anti-Inflammatory

Tea-tree oil thanks to its main component, terpinen-4-ol, can reduce histamine-induced skin inflammation as demonstrated in a study with 27 volunteers [164]. Javed *et al.* [165] reviewed the role of dentifrices with essential oil formulations in periodontal healing retrieved from 20 clinical trials from 1968 to 2010 and concluded that dentifrices formulated with essential oils have beneficial effects on the clinical and microbiological parameters of periodontal inflammation.

Oral lichen planus is a chronic inflammatory condition that affects mucous membranes of the mouth. A randomized, double-blind, clinical trial involving 40 patients comparing the benefits of using aloe vera versus triamcinolone acetonide (a topical corticosteroid) demonstrated that aloe vera gel was more effective than the corticosteroid in the treatment of oral lichen planus [166].

Lalicevic and Djordjevic [167] compared in an open-label, single-blind, randomized clinical trial benzydamine hydrochloride (BNZD) and *Salvia officinalis* (SO) as adjuvants applied locally to a systemic nonsteroidal anti-inflammatory drug (ibuprofen for children and diclofenac in adults) in controlling pain after tonsillectomy, adenoidectomy, or both. In this study, they showed that the risk for severe pain after tonsillectomy, adenoidectomy, or both was reduced when BNZD was used as adjuvant therapy instead of SO. Also there was a lower infection risk when BNZD, rather than SO, was applied as adjuvant therapy.

### 4.3. Regenerative

Healing advances were observed in a clinical trial with 120 primiparous women, with singleton pregnancy, who were treated during episiotomy recovery either with lavender oil or with povidone-iodine (controls). The results concluded that there was no significant difference between those two groups in surgery-site complications. However, redness in lavender group was significantly less than in controls [168]. Another clinical trial with 89 women was carried out to analyze the recovery after episiotomy using lavender based-on olive oil and olive oil [169]. The results also suggested that lavender based-on olive oil and olive oil should be added to routine water sitz bath for post-episiotomy care. In another clinical trial involving 111 primiparous women, Eghdampour *et al.* [170] demonstrated that women treated either with aloe vera or with calendula ointment showed a faster episiotomy wound healing compared to the untreated group.

A clinical study involving 30 patients treated with sesame oil or just with saline having fresh traumatic wounds showed that this oil was effective by reducing pain, minimizing wound surface, reducing the discharge and promoting the epithelialization compared to the controls [171].

Aloe vera gel has been traditionally used to treat burn wounds and several studies concluded that cumulative evidence tends to support that aloe vera might be an effective interventions used in burn wound healing for first to second degree burns but the same study states that more studies are needed to corroborate it [172]. A systematic review of the literature including clinical trials concluded that controlled clinical trials in humans demonstrated no benefit when aloe vera was incorporated into topical therapy [173].

A prospective randomized double-blind clinical trial was conducted with 90 women who had undergone cesarean operation applying aloe vera or just a simple dressing. A significant difference was observed between the two groups with respect to the wound healing score 24 h after the operation; however after eight days, the difference in the wound healing score was not significant [174].

A prospective clinical trial was conducted with 60 patients to evaluate the effects of a topical cream containing 0.5% aloe vera juice powder in the treatment of chronic anal fissures by Rahmani *et al.* [175]. The study concluded that there were statistically significant differences in chronic anal fissure pain, hemorrhaging upon defection and wound healing before and at the end of the first week of treatment with the gel in comparison with the untreated group (placebo). A comparative study stablished between second degree burn patients (50) treated with aloe vera gel compared with those treated with 1% silver sulphadiazine cream showed that faster wound healing and less pain were reported for the group treated with the aloe [176]. Once again we found controversial results or incomparable results because the effect of any antiseptic depends on the wound type, chronicity, the moment to apply the therapeutic compound, the condition of the patients, origin, age, combinatory effects, contact time, the antiseptic of choice for a particular (e.g., bacterial strain) infection, *etc*.

Propolis, a resinous mixture that honey bees collect from tree buds, sap flows, or other botanical sources, was also used in a clinical trial to study its influence in the treatment of recurrent aphthous stomatitis, a common, painful, and ulcerative disorder of the oral cavity [177]. The results obtained indicated that patients in the propolis group self-reported a significant improvement in their quality of life and a statistically significant reduction of outbreaks compared to the untreated controls.

A randomized clinical trial of 37 patients with neuropathic diabetic foot ulcers was set to analyze the benefits of extract of kiwifruit compared to a standard dressing treatment [178]. The group treated with the kiwifruit experienced a larger reduction in the size of the ulcer than the untreated one at the same time. The amount of collagen, angiogenesis, vascularization and granulation tissues were significantly higher in the experimental groups than in the controls. Wound healing kinetics were also evaluated on 34 patients with chronic venous leg ulcers treated either with the plant species *Ageratina pichinchensis* or with 7% propylene glycol alginate [179]. The results showed that the plant extract produced a 100% therapeutic effectiveness, while the control treatment achieved this condition in 81.8% of the control group patients. Ulcer size reduction was significantly higher in the group of patients administered with the extract.

Banana leaf, tree bark, cocoa, turmeric, β-glucans, *etc.* have also demonstrated their antioxidant, anti-inflammatory, antimicrobial, and angiogenic properties in *in vitro* studies and, for some of them, in clinical trials [180].

There is an endless list of natural origin products which have been used since antiquity to heal wounds, but still scientific evidence of those properties is still to be demonstrated using double-blinded multi-center randomized placebo-controlled trials to reach a sufficient level of evidence.

### 4.4. Dose Analysis

To compare the doses needed to obtain the same output in the prevention of infection during wound healing when using natural origin components or synthetic ones we compared the minimal inhibitory and minimal bactericidal concentrations (mic and mbc, respectively) published for just honey in the sake of concision. In this regard Tan *et al.* [181] calculated the mics for two different honeys against nine different bacteria (*S. pyogenes*, *coagulase-negative Staphylococci*, *MRSA*, *Streptococcus agalactiae*, *S. aureus ATCC 33591*, *Proteus mirabilis*, *Shigella flexneri*, *Escherichia coli* and *Enterobacter cloacae*) and the values ranged from 8.75% (w/v) to 25% for tualang honey, while those for manuka honey ranged between 8.75% and 20% (w/v). The mics and mbcs reported by Henriques *et al.* [182] when challenging *S. aureus* NCTC to manuka honey were 2.9% (w/v) and 4.5% (w/v) as mean mic and mbc values, respectively. Against *P. aeruginosa* ATCC 27,853 the doses required of manuka honey were 9.5% (w/v) and 12% (w/v), mic and mbc respectively.

Mic and mbc doses of 20% (w/v) and 40% (w/v), respectively were required when using manuka honey against biofilm forming strains of *S. pyogenes* MGAS6180 [183]. Against oral bacteria (*Streptococcus mutans* adhered on a glass substrate), Badet and Quero [184] reported a total inhibition of multi-species biofilm at the concentration of 200 μg/mL using manuka honey and a total biofilm inhibition was reached at a concentration of 500 μg/mL. Propolis was much more effective against *S. aureus* and *Escherichia coli* compared with honey as reported by Rahman *et al.* [185]. These authors estimated that propolis at concentrations of 2.74 to 3.5 and 3.5 mg/mL is effective to inhibit *S. aureus* and *E. coli*, respectively. On the contrary, honey was effective to inhibit *S. aureus* at the concentration of 375 mg/mL but failed to inhibit *E. coli* growth at same concentration.

Different Malaysian honeys were evaluated against *S. aureus*, *Bacillus cereus*, *E. coli*, and *P. aeruginosa* and compared using equivalent phenol concentrations (EPC) [186]. The results showed that gelam honey possessed lowest mic value against *S. aureus* with a 5% (w/v) mic and mbc of 6.25% (w/v). The highest mic values were shown by pineapple honey against *E. coli* and *P. aeruginosa* as well as acacia honey against *E. coli* with a 25% (w/v) mic and a 50% (w/v) mbc values. Agar inhibition assay showed kelulut honey to possess highest total antibacterial activity against *S. aureus* with 26.49 EPC and non-peroxide activity of 25.74 EPC. Lowest antibacterial activity was observed in acacia honey against *E. coli* with total activity of 7.85 EPC and non-peroxide activity of 7.59 EPC.

The antimicrobial efficacy of conventional antiseptics has been reported against *S. aureus* (including MRSA), *Enterococcus faecalis* (including vancomycinresistant *Enterococcus*), *Streptococcus pneumoniae*, *E. coli*, *P. aeruginosa*, *Clostridium perfringens*, *Haemophilus influenzae* and *Candida albicans* using PVP-iodine, triclosan, chlorhexidine, octenidine and polyhexanide as conventional antiseptics used in wound treatment [187]. The results showed that for chlorhexidine, octenidine and polyhexanide, mic_48_ and mbc_24_ ranged from 16 to 32 mg/L. Maximum values for triclosan ranged from 256 to 512 mg/L, with an efficacy gap against *P. aeruginosa*, while the maximum values of PVP-iodine were 1024 mg/L, with a gap against *S. pneumoniae*. Comparing the minimal effective concentrations, octenidine was the most effective. After 1 min, only octenidine and PVP-iodine fulfilled the requirements for antiseptics. We can conclude that, according to those reported data, honey shows mic and mbc values in the same order of magnitude than conventional antiseptics and potentially applicable after validation in clinical settings. As an example, commercially available dressings based on chemically treated honey are nowadays available in Europe [188].

## 5. Combination of Nanostructured Dressings with Essential Oils

As we mentioned in the previous section, it was already demonstrated that some naturally occurring molecules have antimicrobial [137,151,153], anti-inflammatory [164,166,167] and regenerative properties [172,173,174,175,176,177,178]. From a fabrication point of view, besides the advantages already mentioned of these natural origin materials over antibiotics, their hydrophobicity allows an efficient combination with polymeric matrices often used as wound dressings [189]. Several strategies were used to load essential oils in dressings; the first is the modification of commercial wound dressing by immersion in active principle solutions; the second is the preparation of polymeric films containing those essential oils and the third one is their encapsulation into the polymeric fibers. 

### 5.1. Modification of Commercially Available Wound Dressings

As previously mentioned (Section 4.1), honey impregnated dressings were tested by different researchers with controversial results [139,141,142,143,146], suggesting that the benefits of adding honey to dressings to reduce the rates of wound infection deserves further research.

Edwards-Jones *et al.* [38] impregnated aliquots of tea tree (*Melaleuca alternifolia*), patchouli (*Pogostemon cablin*), lavender (*Lavendula officinalis*), geranium and commercial grapefruit extract (Citridal™) and their combinations on the central area of either a gauze or Gamgee^®^ dressing. The activity of individual oils and combinations against *S. aureus* were studied in an *in vitro* dressing model. Results showed that Geranium and Citricidal™ was the most effective combination against MRSA strains in the vapor phase, but this combination was not as effective against Oxford Staphylococcus. More recently this *in vitro* study was moved towards clinical trials by Chin *et al.* [190]. These researchers used the same dressing model with patients who had wounds infected with *S. aureus*. They found that all participants treated with tea tree oil, except one, showed accelerated healing times. The use of this essential oil in wound dressings appears to be a safe complementary treatment of abscessed wounds. These results suggest that there is, in this case, a correlation between *in vitro* models and *in vivo* scenarios. Since this was a small study (only 10 volunteers) a larger study controlling as many variables as possible is needed as we mentioned in our previous section.

**Figure 6:**
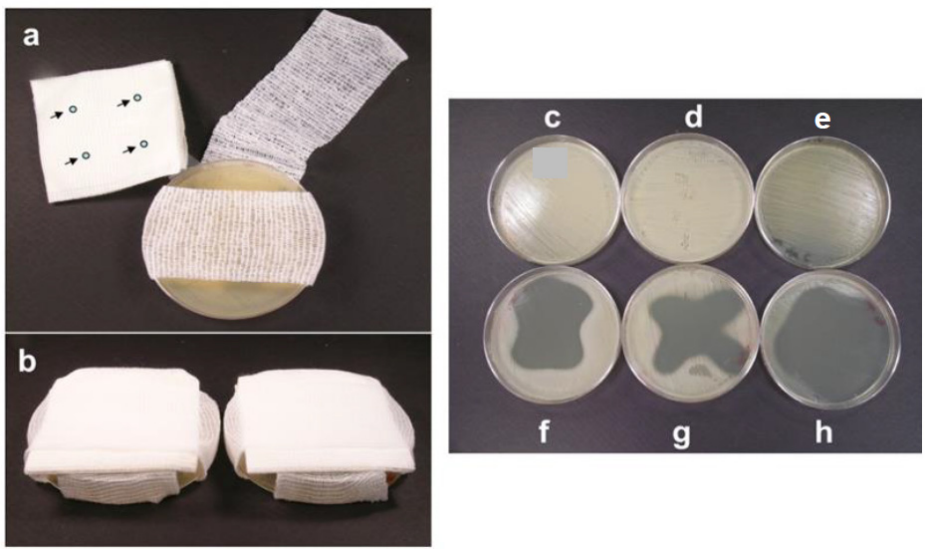
Dressing model using an agar plate. (**a**) arrows indicate the cold pressed Valencia orange oil spots on gauze dressing pad; (**b**) complete setup of dressing model with gauze dressing pad wrapped with bandage. Untreated control plates of bacterial strains *S. aureus*: (**c**) COL; (**d**) Mu50; and (**e**). Inhibition of *S. aureus*: (**f**) COL; (**g**) Mu50; and (**h**) caused by the cold pressed Valencia orange oil [191]. (Copyright BioMed Central 2012).

The effect of Valencian orange oil against MRSA was also evaluated in a dressing model [191]. The orange oil was spotted into cotton dressing pads placed on Petri dishes seeded with MRSA and wrapped with a bandage as shown in Figure 6. The growth of MRSA strains was inhibited by the vapor released from the dressing pad and exhibited clear inhibition zones on agar plates (Figure 6f–h) demonstrating that essential oils can be applied as natural origin anti-MRSA agents on the outer layer of a dressing.

Budzynska *et al.* [192] modified the commercial dressing Sorbact^®^ by its immersion in an essential oil solution (*Melissa citrate indica*, *Pelargonium graveolens*, *Cymbopogon nardus* and *Eugenia caryophyllata*) to evaluate its antimicrobial action. The dressing containing essential oils could keep absorbed bacteria/fungi inside and efficiently limit their growth. As we mentioned before, in order to avoid the problem of the high volatility of the essential oils and to improve their stability they were combined with magnetite nanoparticles (MNP) [135]. The MNP functionalized with the essential oil of *Anethum graveolens* (AG) and SO were added to sterile rayon/polyester based wound dressing by immersion in a chloroform suspension and posterior drying. Both essential oils showed a significant decrease in *C. albicans* viable cell counts; they preferentially inhibited the early stages of biofilm formation (after 24 h) but also reduced the formation and development of mature biofilms. Due to the different release profiles of the two oils, the inhibitory effect of AG reached a maximum at 48 h while SO reached the maximum at 72 h. Both essential oils increased the resistance of the dressing to fungal colonization. The same group also proved that *Satureja hortensis* essential oil attached to MNP added to commercial dressing exhibited antimicrobial properties being more resistant to *C. albicans* fungal adherence and biofilm development [193]. Holban *et al.* [194] also had used magnetite nanoparticles to stabilize carvone, the major active compound found in *Anethum graveolens* essential oil. Textile wound dressings containing these functionalized nanoparticles significantly reduced microbial adherence and fungal mature *Candida tropicalis* biofilm formation for 72 h.

Other strategies should be implemented to reduce the high volatility of the essential oils which might lead to a burst release of the antibacterial compounds. A sustained long-term release is necessary in order to employ these dressings during the healing process of different wounds and/or at different wound stages.

### 5.2. Polymer Films and Foams Containing Essential Oils

Another way to fix those essential oils to the dressings is their incorporation into polymeric films to prevent their evaporation and burst release. The load of the bioactive species into the films could be performed in a very simple way by casting a solution of the polymer and the essential oil choosing the appropriated surface chemistry to adsorb or chemisorb the essential oil into the polymer.

In this regard, thymol, a monoterpene of the essential oil from *Lippia gracilis*, was mixed in a collagen solution in order to obtain a collagen-based containing thymol film. The dispersion was then casted and allowed to dry [195]. Sterilized pieces of the obtained films were tested to evaluate their anti-inflammatory effects on rats and mice. The results revealed that thymol significantly ameliorated inflammatory responses and possessed wound healing potential. The films promoted wound contraction in only 7 to 14 days. Moreover, thymol induced a more regular and dense collagen arrangement of the granulation tissue fibrils/fibres, suggesting that this molecule might also favor fibroblastic proliferation and collagen deposition.

Thymol was also used to load gelatin films to examine their antioxidant and antimicrobial action [196]. In this case, the films were also prepared by casting gelatin, glycerol as plasticizer, glutaraldehyde as cross-linker and thymol. The resulting gelatin films showed good mechanical properties and the presence of this natural origin monoterpene also increased the solubility and reduced the swelling and water uptake. Gelatin films incorporated with thymol exhibited excellent antioxidant and antibacterial properties. However, the same authors pointed out that further *in vivo* research is needed.

Chitosan is another polymeric matrix that has been used to host thyme oil. Altiok *et al.* [197] incorporated this essential oil into chitosan films by solvent casting. Chitosan was selected because of its demonstrated nontoxicity, biodegradability, biofunctionality, biocompatibility and depending on its deacetylation degree and molecular weight, antimicrobial characteristic. Tensile strength and Young’s modulus were reduced by the incorporation of thyme oil because of the resulting porous structure after the addition of the oil. But this porosity also leads to higher values of water vapor permeability and higher oxygen diffusion, properties that, as we mentioned before, are desirable during the wound healing process to maintain the moisture balance. The antimicrobial activity of the films was evaluated against four different pathogens: *E. coli*, *Klebsiella pneumoniae*, *P. aeruginosa* and *S. aureus*. The highest antimicrobial effect was achieved against *K. pneumoniae* at 1.2% (v/v) of thyme oil being the minimum concentration which prevents the growth of all selected microorganisms. Those films also showed antioxidant activity, although further studies are necessary to determine the cytotoxicity of the prepared films in order to confirm the potential of thymol loaded chitosan films as wound dressing materials.

Sodium alginate (NaAlg) is also preferred as a construction material for wound dressing applications since it has shown wound healing properties [198]. Liakos *et al.* [199] blended *Elicriso italic*, chamomile blue, cinnamon, lavender, tea tree, peppermint, eucalyptus globulus juvenile, lemongrass, and lemon essential oils with NaAlg solutions containing also glycerol as plasticizer. Igepal, as surfactant, was also added to the solution in order to control and minimize the size of the highly hydrophobic essential oil. The resulting solutions were then cast onto glass slides to obtain the films. The antibacterial and antifungal activities of the prepared films were tested against *E. coli* and *C. albicans*. Cinnamon, tea tree, peppermint and lemongrass loaded films were able to inhibit the growth of *C. albicans* at all the concentrations studied (15%, 50% and 66%) and they were able to inhibit *E. coli* at the intermediate and highest concentrations. Lavender was able to inhibit both microorganisms at the intermediate and highest concentrations. Eucalyptus and lemon were incapable to stop the *E. coli* growth but lemon successfully inhibited the growth of *C. albicans* in all concentrations and eucalyptus in the intermediate and highest ones. Elicriso oil was found to be effective against fungal colonization only at the highest used concentration and chamomile blue was the only essential oil which was found to be ineffective against both *E. coli* and *C. albicans* in all the studied concentrations.

Another polymer used to prepare films loaded with essential oil is PVA because this polymer shows excellent mechanical and barrier properties. Kavossi *et al.* [200] prepared PVA films incorporated with Zataria multiflora essential oil by casting. The resulting films showed antifungal activity against *Paecilomyces variotii*, *Trichoderma harizanum*, *Aspergillus oryzae*, and *Aspergillus niger*. The authors claimed that this oil is a good source of phenolic monoterpenes (thymol and carvacrol) that easily interfere with the phospholipids present in the cell membranes.

The solution casting method to obtain polymeric films loaded with essential oils even if it is a relatively simple method can have some drawbacks including the use of organic solvents, low incorporation yields and resulting in heterogeneous dispersions. Dias *et al.* [201] proposed supercritical solvent impregnation to overcome most of these limitations. The use of supercritical carbon dioxide avoids the use of organic solvents and allows the possibility to work at low temperatures and with most of the polymeric matrices usually intended for wound dressings. Using this method they added quercetin and thymol as bioactive compounds to natural-based polymeric matrices: *N*-carboxybutylchitosan (CBC) and agarose (AGA). CBC films and foams of both polymers were prepared by solvent casting and freeze drying, respectively. These foams and films were loaded with a high pressure mixture of bioactive compound supercritical CO_2_ and a co-solvent (ethanol). The *in vitro* study revealed that all quercetin/thymol was released during a 9 h period. As expected, the higher porosities of foam-like structures permitted the loading of higher amounts of the essential oils and an increased available surface area. The systems presented adequate water vapor adsorption and transmission rates that were in the range required during wound healing.

### 5.3. Fiber-Based Mats

Wound dressings composed of polymer woven or non-woven microfibers or nanofibers (natural origin and synthetic) have received significant interest because of their outstanding characteristics [202,203] but just a few of the reported materials incorporate essential oils in their formulation.

Alginate fibers produced by wet spinning were loaded with eucalyptus essential oil by doping the spinning solution of alginic acid with the bioactive compound [204]. Divalent cations (Ca^2+^) were used in the coagulation bath to cross link the alginic acid polymer chains by the formation of junction zones (egg-box model). The antibacterial activity of the resulting fibers was confirmed against *S. aureus*.

**Figure 7:**
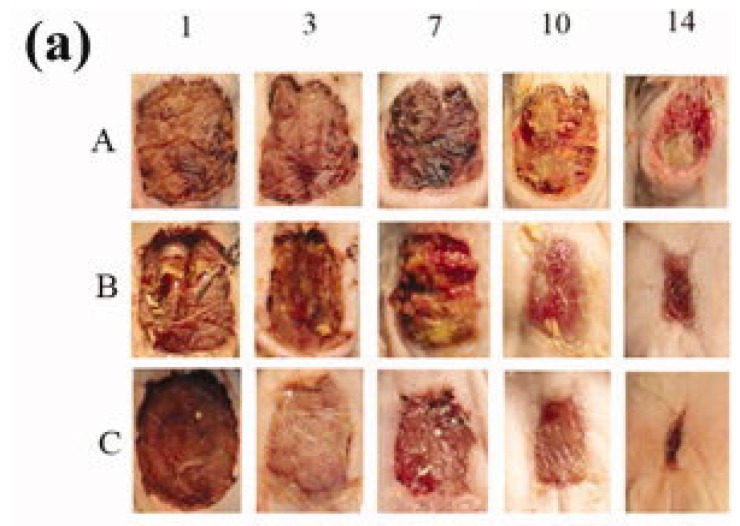

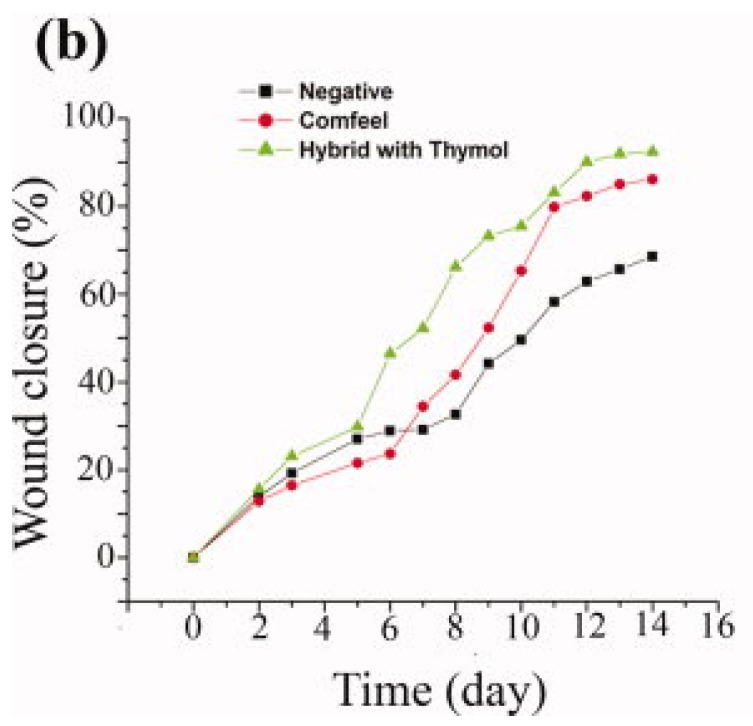
(**a**) Photographs obtained from wounds covered with (A) gauze, (B) Comfeel^®^ Plus, and (C) electrospun PCL/PLA (50/50) nanofibrous mats containing thymol at different times: 1, 3, 7, 10, and 14 days. (**b**) Degree of closure (%) for wounds treated with gauze, Comfeel Plus, and electrospun PCL/PLA (50/50) nanofibrous mats containing thymol after days of post-treatment [205]. (Copyright Jonn Wiley & Sons 2013)

PCL and PLA nanofibrous mats loaded with thymol were prepared by Karami *et al.* [205] by dissolving the polymers in a mixture of chloroform and dimethylformamide together with thymol. The authors found that thymol acts as plasticized and rearranged the polymer chain layer leading to a decrease in the viscosity of the polymer solution and, as a consequence, the average nanofiber diameter was reduced. Due to the limited solubility of thymol in the electrospun polymer solutions only 1.24% v/v of herbal drug could be loaded in the fibers. A bimodal release profile was observed: in the first 12 h there was a burst release, following by a gradual drug release observed up to 48 h. The antibacterial evaluation of the hybrid mat carried out via the disc diffusion method resulted in the formation of inhibition zones of 10.4 and 7.8 mm against *S. aureus* and *E. coli*, respectively. The performance of the PCL/PLA mat containing thymol in an *in vivo* rat wound healing model showed a faster healing than when using a Comfeel^®^ Plus commercial dressing. This improvement in the healing process was attributed to the water vapor permeability of the electrospun mats (3–4 mg/cm^2^h^−1^) compared to the not permeable commercial material (Figure 7).

Cinnamon, lemongrass, and peppermint essential oils were encapsulated in electrospun cellulose-based nanofibers [189]. Solutions of the cellulose acetate and the essential oils in acetone were electrospun to obtain the loaded fibers. Antimicrobial assays using *E. coli* demonstrated that fibers encapsulating 6.2 and 25 wt. % of essential oils were able to effectively stop upon contact the proliferation of *E. coli*. The comparison of the bactericidal activity of the resulting fiber mats versus planar films showed that the fibrous network strongly enhances the antibacterial effect against *E. coli* (~1.5 µm in diameter) [200]. However, against *C. albicans* (~4 µm in diameter), even with a 40 w/w % of essential oils in the cellulose acetate solution was not capable to successfully inhibit their growth. According to the authors, *E. coli* was able to penetrate inside the fiber matrix (empty space around 2–10 µm) and get in direct contact with the antibacterial material, while *C. albicans* cells did not penetrate inside the mesh of the mat and therefore they were only in contact with the outermost layers. The biocompatibility of the mats was evaluated using two *in vitro* cell models: immortalized fibroblasts and normal human keratinocytes. The unloaded fibers mats exhibited high cell compatibility and no cytotoxicity at the doses tested and the morphological analysis revealed that the cells can attach and spread on the fibers’ surface. But a reduction in the cell viability was observed for essential oil loaded fibers due to the anti-proliferative effect of the compounds against eukaryotic cells [206,207].

## 6. Conclusions

Wounds are undoubtedly a severe problem for patients’ health and quality of life as well as for the health care system. The chronicity of a wound is usually derived of previous pathologies which hamper wound healing. The basic concept of tissue, infection/inflammation, moisture, and edge of wound remains as the best care in order to speed up the natural healing process. Nowadays, wound care practitioners are aware of the importance of both wound examination and assessment of the general health status of the patient to achieve successful wound healing.

Topical wound dressings play an important role in the wound management and their therapeutic availability has increased remarkably in the past years. However, in spite of wide variety of existing dressings (passive, interactive and bioactive) on the market, there is not any wound dressing that fulfills all the requirements to be applied in the different stages of wound healing. Therefore, the search for an ideal dressing continues to be a challenge in the tissue engineering field. Novel strategies in wound repair are currently centered on the development of 3D scaffolds with structural and biochemical similarity to the natural ECM. Nanotechnological advancements have allowed creating materials and devices that are overcoming some of the limitations of the current dressings. Thus, nanofibrous scaffolds made of natural origin and/or synthetic polymers hold great potential for wound dressings because of their unique properties such as high surface area to volume ratio, adequate porosity, and biocompatibility. Electrospinning has become the most used technique for the fabrication of nanodressings due to its simplicity and versatility. The loading of different types of therapeutic compounds such as drugs, biomacromolecules or other natural origin compounds with antimicrobial, anti-inflammatory or regenerative properties in those nanofibers is attracting much attention because of the possibility to improve the current bioactive dressings with the perspective of achieving better therapeutic alternatives to non-healing wounds, which also might fulfil the reduction of adverse side effects and the protection against wound infection.

Microbially compromised wounds have been treated with different essential oils, honey, cationic peptides, aloe vera, plant extracts, and other natural occurring antimicrobial, anti-inflammatory, and regenerative molecules but the available evidence is limited and insufficient to be able to draw reliable conclusions and to extrapolate those findings to the clinical practice. Instead of talking about the beneficial or inert effects of those natural occurring materials the scientific community directs towards the identification of the main active components involved and their mechanism of action during the healing, antimicrobial, or regenerative processes and in carrying out systematic and comparative controlled tests. For many of those components the scientific basis behind which support the beneficial effect at a molecular level still needs to be addressed. The evidence and some promising preliminary results indicate that future comparative studies are justified. However, some of the clinical trials reported are biased due to the lack of methodology during the tests. Also, several factors that have not been, in many cases, previously considered should be taking into account in those future studies including the healing kinetics, the wound type, chronicity, the moment to apply the therapeutic compound, the condition of the patients, origin, age, combinatory effects, contact time, the bacterial strain present, *etc.* Double-blinded multi-center randomized placebo-controlled trials are needed to reach a sufficient level of evidence and to generate new practices to extrapolate those findings to the clinical practice.

The modification of commercial dressing with essential oils and honey has proven to provide them with bactericidal and antifungal properties that would favor the wound healing process. However, these materials cannot solve the problem of the high volatility of the EOs that severely limit the effectiveness of the dressing. Some attempts to increase the stability of EOs were carried out using magnetic nanoparticles to anchor them to the dressing showing good results. A more effective way to control the releasing of the bioactive compounds necessary to the healing process of different wounds and/or at different wound stages would be the encapsulation in the polymeric matrix constituting the dressings.
